# Ingestion of radioactively contaminated diets for two generations in the pale grass blue butterfly

**DOI:** 10.1186/s12862-014-0193-0

**Published:** 2014-09-23

**Authors:** Chiyo Nohara, Wataru Taira, Atsuki Hiyama, Akira Tanahara, Toshihiro Takatsuji, Joji M Otaki

**Affiliations:** BCPH Unit of Molecular Physiology, Department of Chemistry, Biology and Marine Science, University of the Ryukyus, Okinawa, 903-0213 Japan; Instrumental Research Center, University of the Ryukyus, Okinawa, 903-0213 Japan; Graduate School of Fisheries Science and Environmental Studies, Nagasaki University, Nagasaki, 852-8521 Japan

**Keywords:** Fukushima nuclear accident, Ingestion, Internal exposure, Low-dose exposure, Pale grass blue butterfly, Radioactive contamination, Transgenerational effect

## Abstract

**Background:**

The release of radioactive materials due to the Fukushima nuclear accident has raised concern regarding the biological impacts of ingesting radioactively contaminated diets on organisms. We previously performed an internal exposure experiment in which contaminated leaves collected from polluted areas were fed to larvae of the pale grass blue butterfly, *Zizeeria maha*, from Okinawa, which is one of the least polluted localities in Japan. Using the same experimental system, in the present study, we further examined the effects of low-level-contaminated diets on this butterfly. Leaves were collected from two localities in Tohoku (Motomiya (161 Bq/kg) and Koriyama (117 Bq/kg)); two in Kanto (Kashiwa (47.6 Bq/kg) and Musashino (6.4 Bq/kg)); one in Tokai (Atami (2.5 Bq/kg)); and from Okinawa (0.2 Bq/kg). In addition to the effects on the first generation, we examined the possible transgenerational effects of the diets on the next generation.

**Results:**

In the first generation, the Tohoku groups showed higher rates of mortality and abnormalities and a smaller forewing size than the Okinawa group. The mortality rates were largely dependent on the ingested dose of caesium. The survival rates of the Kanto-Tokai groups were greater than 80%, but the rates in the Tohoku groups were much lower. In the next generation, the survival rates in the Tohoku groups were below 20%, whereas those of the Okinawa groups were above 70%. The survival rates in the second generation were independent of the locality of the leaves ingested by the first generation, indicating that the diet in the second generation was the determinant of their survival. Moreover, a smaller forewing size was observed in the Tohoku groups in the second generation. However, the forewing size was inversely correlated with the cumulative caesium dose ingested throughout the first and second generations, indicating that the diet in the first generation also influenced the forewing size of the second generation.

**Conclusions:**

Biological effects are detectable under a low ingested dose of radioactivity from a contaminated diet. The effects are transgenerational but can be overcome by ingesting a non-contaminated diet, suggesting that at least some of the observed effects are attributable to non-genetic physiological changes.

## Background

The collapse of the Fukushima Dai-ichi Nuclear Power Plant (NPP) due to the Great East Japan Earthquake on 11 March 2011 resulted in the release of a massive amount of radioactive materials into the surrounding environment. This large-scale environmental pollution has impacted the lives of organisms living in the polluted areas. A field study revealed that the number of butterflies decreased in the polluted areas [1,2], and morphological abnormalities have been detected in aphids from Fukushima [3]. However, such studies examining the biological impacts of the accident are still scarce.

Our group has been studying the development and evolution of butterflies in response to environmental stress [4]. One of our favourite butterflies is a small lycaenid butterfly, the pale grass blue, *Zizeeria maha* (Lepidoptera, Lycaenidae), which is found throughout Japan, except in Hokkaido [4-6]. Using this butterfly as an experimental system, we previously reported various morphological abnormalities and a smaller forewing size in adults collected from highly polluted areas [7-9]. We showed that some abnormal traits, including aberrations in the wing colour pattern, were heritable, suggesting genetic damage introduced by the accident [7-9]. We believe that the proposed genetic damage was introduced mainly by the initial exposure of the butterflies immediately after the explosion [9]. We reproduced these field-based results through an external exposure experiment in which butterfly larvae and pupae were raised near an artificial caesium radiation source as well as through an internal exposure experiment in which larvae consumed contaminated host plant leaves collected from polluted areas [7-9]. High mortality and abnormality rates were detected [7], and a small forewing size (i.e., body size) was also observed [7], similar to the outcome of the food stress in butterflies [10].

**Figure 1:**
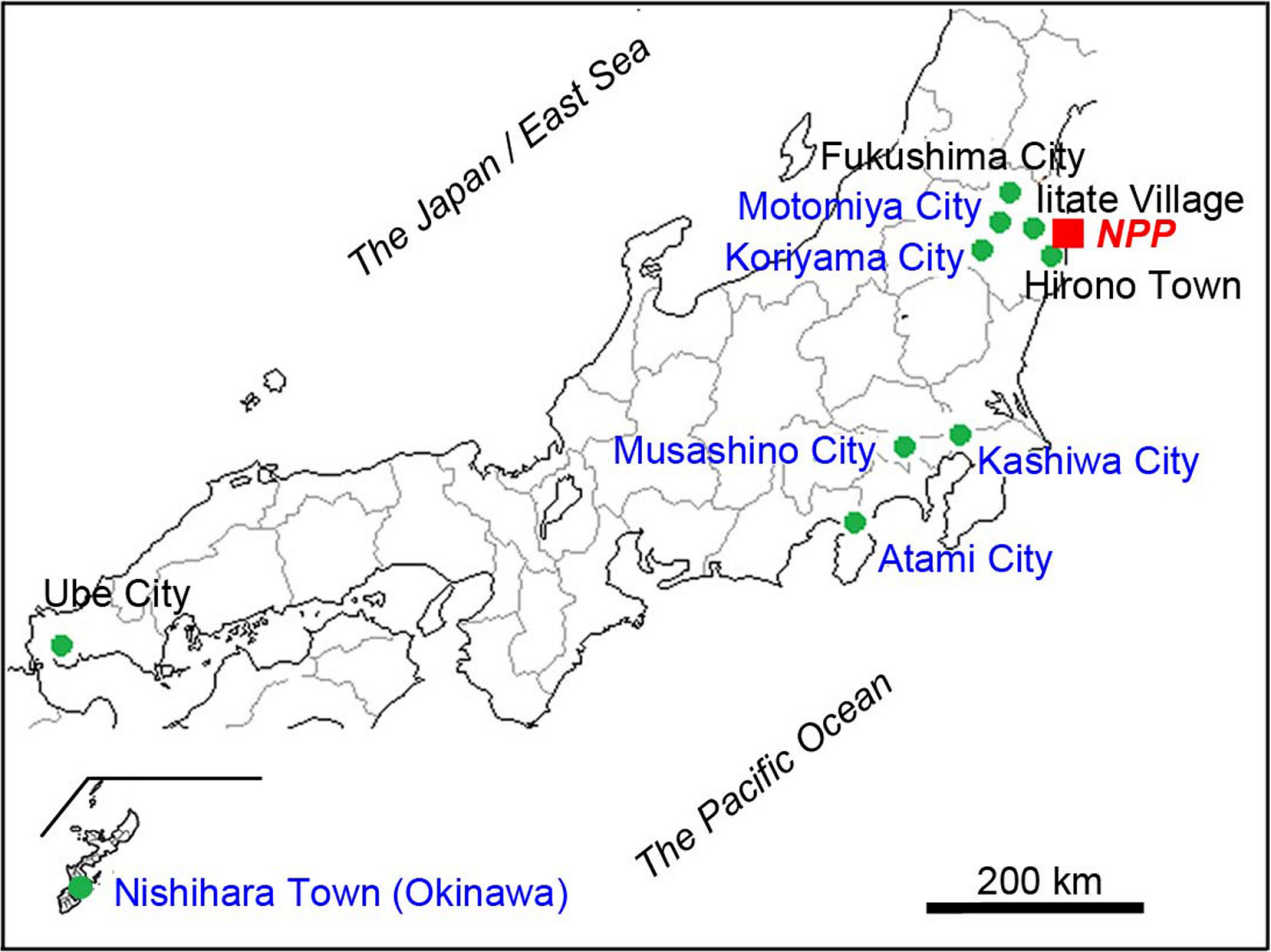
**Collection localities for host plant leaves.** The localities examined in the present study are shown in blue letters and those examined in the previous studies [7-10] in black letters. The Fukushima Dai-ichi Nuclear Power Plant is indicated as NPP in red.

In a previous study, we collected host plant leaves of *Oxalis corniculata* (in the summer of 2011) from four relatively highly polluted localities in the Tohoku district (Iitate-montane, Iitate-flatland, Fukushima, and Hirono), in addition to the control locality, Ube (Figure 1) [7]. We further quantified caesium radioactivity in pupae that ingested contaminated leaves and determined the mathematical relationships between the ingested dose of caesium and the resultant rates of mortality and abnormalities [11]. We found that the mortality and abnormality rates increased sharply under low doses of caesium ingestion, following a power function. However, as the leaves showing the lowest concentration recorded in that study were collected from Hirono (1,452 Bq/kg), leaves from other localities with much lower contamination levels should be examined to better understand the effects of low-level radiation ingestion.

In the present study, we collected leaves from host plants of the pale grass blue butterfly from six localities showing relatively low-level contamination: Motomiya, Koriyama (both in Fukushima Prefecture in the Tohoku district; collected in the fall of 2012), Kashiwa and Musashino, (in Chiba Prefecture and Tokyo, respectively, in the Kanto district; collected in the summer of 2012), Atami (in Shizuoka Prefecture in the Tokai district; collected in the summer of 2012) and Okinawa (one of the farthest districts from the Fukushima Dai-ichi NPP in Japan; collected in the summer and fall of 2012) (Figure 1; Table 1). These host plant leaves were given to larvae from Okinawa, which were not affected by the nuclear accident to any detectable degree, under our standard rearing conditions [12]. We evaluated the biological impacts of various levels of contamination on this butterfly in this manner. Furthermore, we performed the internal exposure experiment not only in the first (F_1_) generation, but also in the second (F_2_) generation to examine possible transgenerational effects.

**Table 1:** **Information for the collection localities for host plant leaves**

	**Collection site**	**Collection date**	**Distance from the NPP [km] (average)**	**Ground radiation [μSv/h] (average)**
Okinawa	1 Senbaru, Nishihara, Okinawa Prefecture, University of the Ryukyus	20, 30 July 2012	1760	0.05 (8 April 2012)
		2-22 October 2012		
Atami	1164 Izusan, Atami City, Shizuoka Prefecture	25 July 2012	311	0.07
		4 August 2012		
Musashino	2 Gotenyama, Musashino City, Tokyo	24 July 2012	232	0.12
		3 August 2012		
		20 October 2012*^1^		
Kashiwa	6 Kashiwanoha, Kashiwa City, Chiba Prefecture	23 July 2012	196	0.47
		7 August 2012		
		5 October 2012*^1^		
Koriyama	Tomita, Koriyama City, Fukushima Prefecture	4, 14 October 2012	61	1.13
		1 November 2012		
Motomiya	Arai, Motomiya City, Fukushima Prefecture	4, 14 October 2012	59	1.42
		1 November 2012		

*^1^Leaves were collected only for activity measurements.

## Results

### Effects on the F_1_ generation

**Figure 2:**
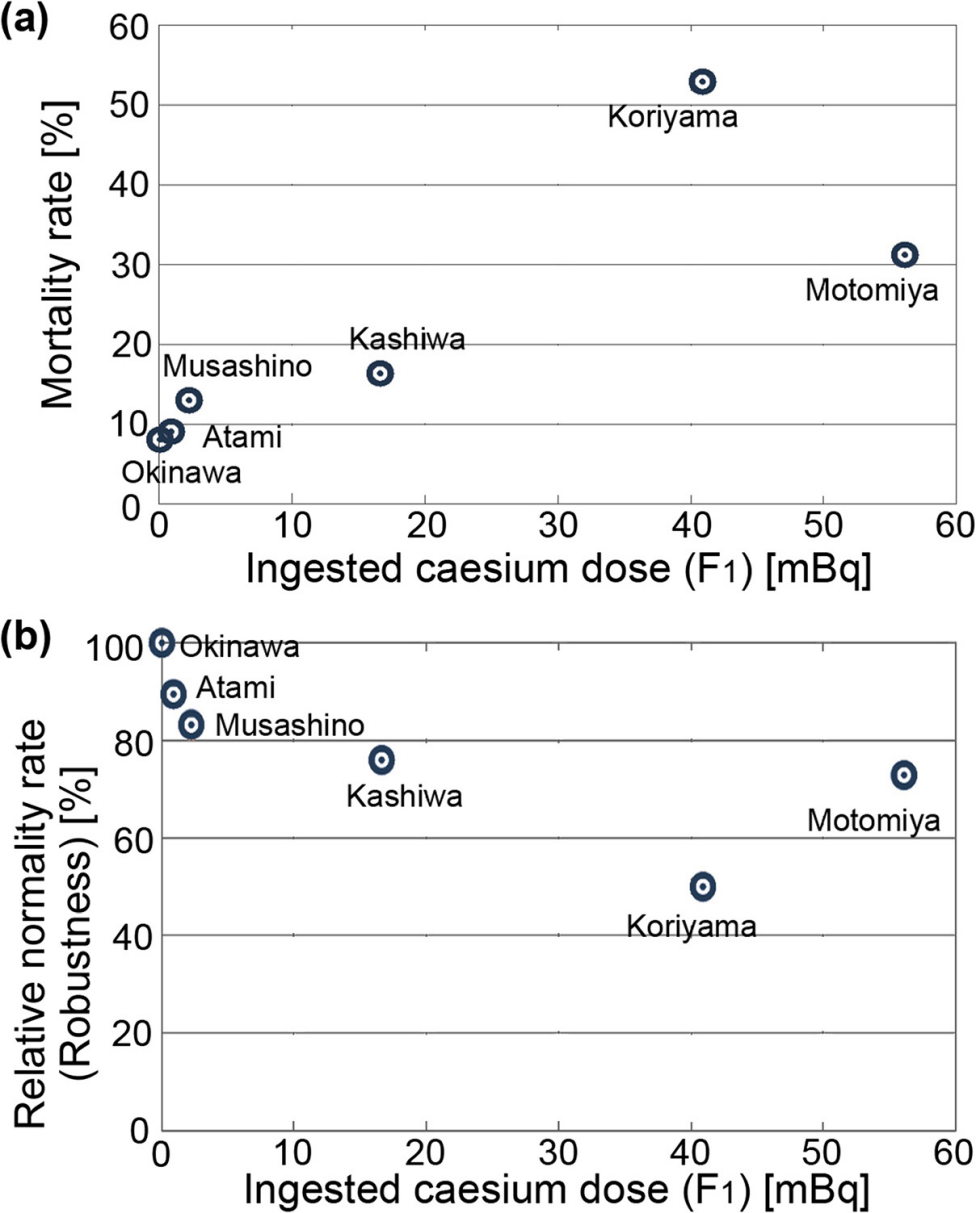
**Effects of the ingested caesium dose on the F**_**1**_ **generation. (a)** Mortality rate. **(b)** Relative normality rate (robustness).

Previous studies showed that an average larva has a body weight of 0.035 g and consumes 0.388 g of leaves throughout its life on average [11]. We obtained the concentrations of caesium activity in leaves collected from the six indicated localities (Table 2), and we calculated the amount of caesium activity ingested by each feeding group of larvae, together with the resultant mortality and abnormality rates (Table 3). The mortality rate and the abnormality rate were defined as percent dead or percent abnormal and dead (and hence, total abnormality rate), respectively. We then examined the dose–response relationship between the ingested caesium dose and the mortality rate (Figure 2a). The mortality rate (*y*) increased linearly in accordance with an increase of the caesium dose (*x*) with the regression model, *y* = 0.60 (±0.21) *x* + 10.21 (±6.23) (*R*^2^ = 0.659, *df* = 5, *F* = 7.71, *p* = 0.0497). Remarkably, the mortality rate of the Koriyama group (40.9 mBq per larva) was 53%. The dose–response relationship for abnormality rates was almost identical to that for mortality rates (not shown).

**Table 2:** Caesium radioactivity in host plant leaves* ^**1**^

	**Activity of** ^**137**^ **Cs (±SE) [Bq/kg]**	**Activity of** ^**134**^ **Cs (±SE) [Bq/kg]**	**Total for** ^**137**^ **Cs and** ^**134**^ **Cs [Bq/kg]**
Okinawa (for F_1_)	0.12 ± 0.02	0.06 ± 0.02	0.18 ± 0.03
Atami (for F_1_)	1.48 ± 0.06	1.05 ± 0.06	2.53 ± 0.08
Musashino (for F_1_)	3.82 ± 0.07	2.56 ± 0.07	6.38 ± 0.10
Kashiwa (for F_1_)	28.40 ± 0.33	19.17 ± 0.32	47.57 ± 0.46
Koriyama (for F_1_)	71.87 ± 0.40	45.34 ± 0.37	117.21 ± 0.54
Motomiya (for F_1_)	98.19 ± 0.54	62.41 ± 0.49	160.60 ± 0.73
Okinawa (for F_2_)	0.11 ± 0.02	0.06 ± 0.02	0.18 ± 0.03
Koriyama (for F_2_)	71.73 ± 0.40	44.07 ± 0.36	115.80 ± 0.54
Motomiya (for F_2_)	98.00 ± 0.53	60.65 ± 0.48	158.65 ± 0.72

*^1^Activity levels were calculated assuming that the larvae ate all of the leaves required for their subsequent growth at once on the first day of ingestion of the contaminated leaves and that ^137^Cs and ^134^Cs were released at a 1:1 activity ratio on 15 March 2011 in a single burst from the Fukushima Dai-ichi NPP.

**Table 3:** **Ingested caesium dose and mortality and abnormality rates in the F**_**1**_ **generation**

**The F**_**1**_ **leaves from:**	**Number of larvae (** ***n*** **)**	**Ingested caesium dose [mBq]**	**Mortality rate [%]**	**Total abnormality rate [%]**
Okinawa	308 (Summer = 67, Fall = 241)	0.0629 ± 0.0011	8.05*^1^	8.25*^1^
Atami	55	0.883 ± 0.003	9.10	9.10
Musashino	54	2.23 ± 0.004	13.0	13.0
Kashiwa	55	16.6 ± 0.02	16.4	16.4
Koriyama	249	40.9 ± 0.02	53.0	54.2
Motomiya	256	56.1 ± 0.03	31.2	32.0

*^1^Mean values for the summer and fall results.

Because surviving adults are not necessarily healthy regarding morphological traits, we calculated relative normality values for adults in reference to the control Okinawa groups as an indication of the robustness of each feeding group (Figure 2b). To this end, the ratio of the number of morphologically normal (non-abnormal) adults to the original number of larvae was calculated, and the ratios in the control Okinawa groups were set at 100%. As expected, the robustness distribution (*y*) appeared to show a non-significant inverse response relationship with the caesium dose (*x*) with the regression model, *y* = −0.53 (±0.24) *x* + 89.07 (±6.23) (*R*^2^ = 0.555, *df* = 5, *F* = 4.99, *p* = 0.089). When the Motomiya sample was excluded as an outlier, the regression equation becomes *y* = −1.03 (±0.17) *x* + 92.37 (±3.41) (*R*^2^ = 0.923, *df* = 4, *F* = 35.95, *p* = 0.0093), showing a highly significant dose–response relationship and being consistent with the results found for the mortality rates.

**Figure 3:**
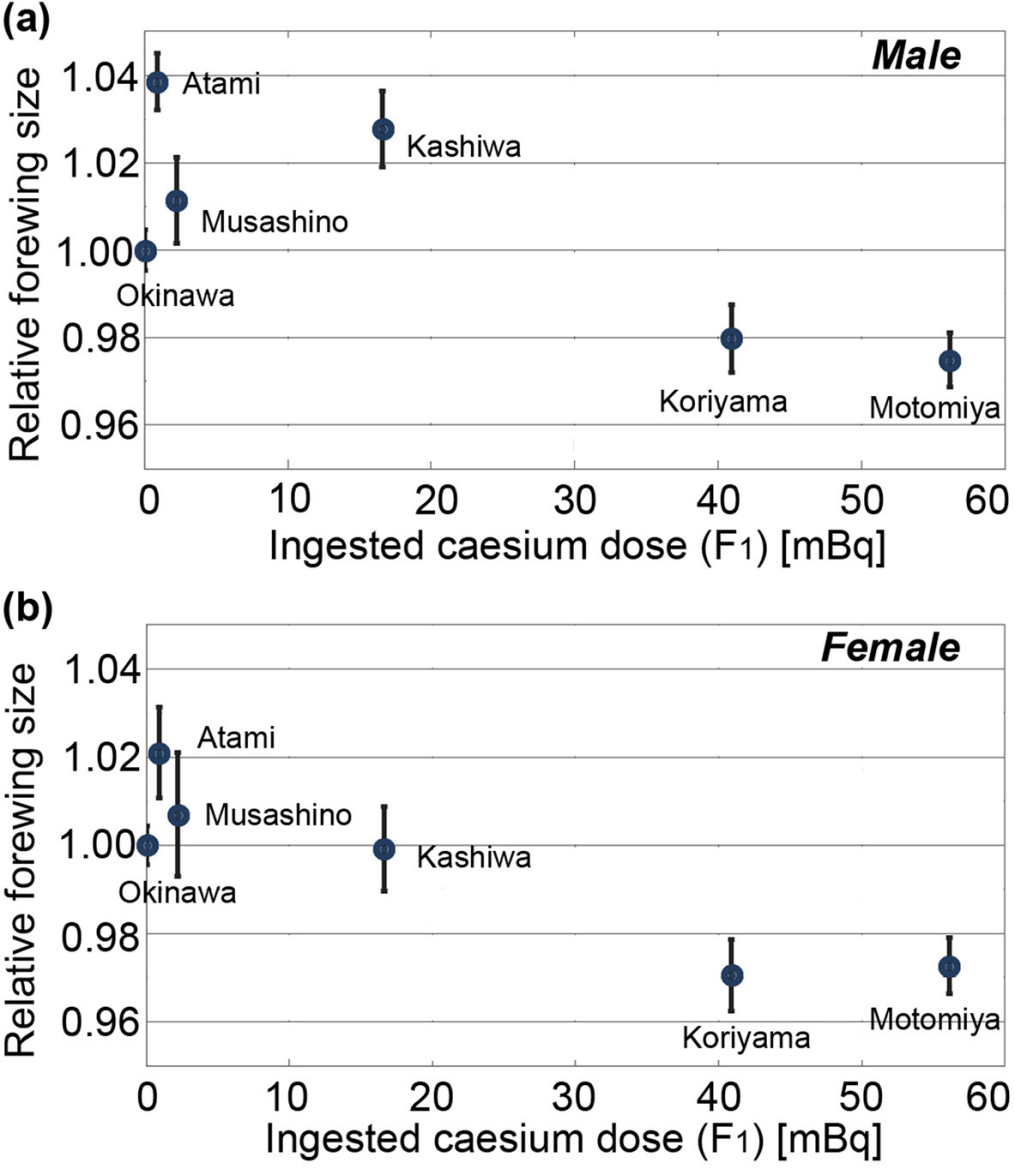
**Relationship between the ingested caesium dose and wing size in the F**_**1**_ **generation.** The relative mean wing size is shown, together with standard error bars. The size in the Okinawa group was set as 1.00. **(a)** Male forewings. The number of individuals that were subjected to measurement is as follows: Okinawa (*n* = 97), Atami (*n* = 25), Musashino (*n* = 19), Kashiwa (*n* = 25), Koriyama (*n* = 84), and Motomiya (*n* = 71). **(b)** Female forewings. The number of individuals that were subjected to measurement is as follows: Okinawa (*n* = 99), Atami (*n* = 23), Musashino (*n* = 27), Kashiwa (*n* = 21), Koriyama (*n* = 63), and Motomiya (*n* = 87).

We measured the forewing size of the surviving adults from each feeding group, and these data were plotted against the ingested caesium dose (Figure 3). The forewing size fluctuated at low doses, but it decreased at relatively high doses in both sexes. Statistically significant differences were obtained between the Okinawa and Koriyama groups both in males (*t* = 2.22, *df* = 137, *p* = 0.028, Welch’s *t*-test) and in females (*t* = 3.17, *df* = 98, *p* = 0.0020, Welch’s *t*-test) and between the Okinawa and Motomiya groups both in males (*t* = 3.31, *df* = 166, *p* = 0.0011, Student’s *t*-test) and in females (*t* = 3.51, *df* = 155, *p* = 0.0006, Welch’s *t*-test). A linear regression model between the ingested caesium dose (*x*) and the forewing size (*y*) did not show statistically significant relationship in males with the equation, *y* = −0.00083 (±0.00034) *x* + 1.0215 (±0.0099) (*R*^2^ = 0.598, *df* = 5, *F* = 5.95, *p* = 0.071), but it showed a significant relationship in females with the equation, *y* = −0.00076 (±0.00017) *x* + 1.0098 (±0.0050) (*R*^2^ = 0.827, *df* = 5, *F* = 19.14, *p* = 0.012). This sexual difference may originate from the simple fact that females have larger wings in size.

**Figure 4:**
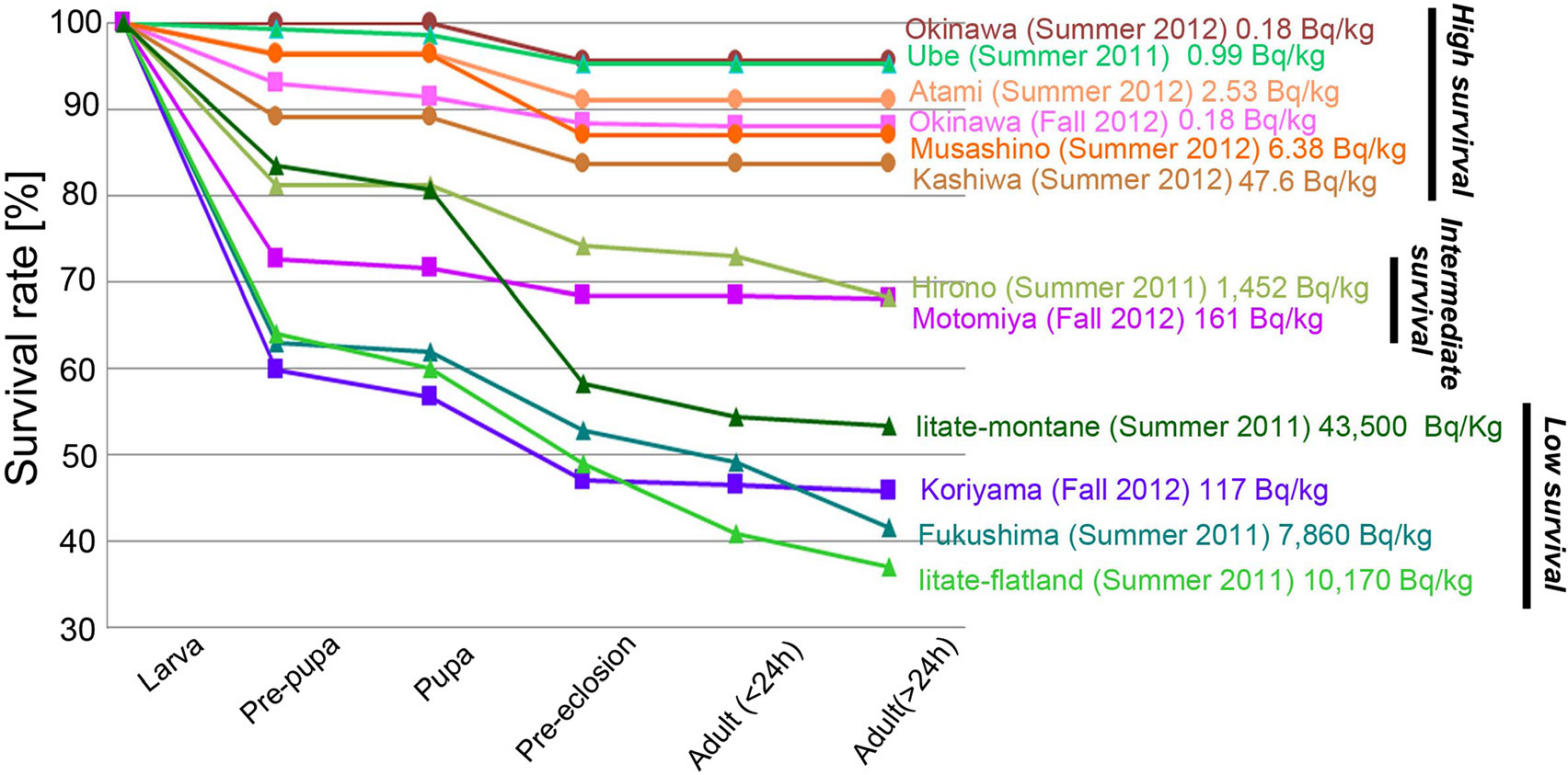
**Survival curves for the F**_**1**_ **generation.** The six groups examined in the present study and the five groups examined in previous studies [7-10] are plotted together. The survival curves were roughly segregated into three clusters of the experimental groups. The concentrations of caesium activity in the ingested host plant leaves are indicated, together with the time period of the experiments. Groups that were examined in the summer of 2012 are indicated by brownish colours with circular symbols. Groups examined in the fall of 2012 are indicated by purplish colours with square symbols. Groups examined in the summer of 2011 in previous studies [7-10] are indicated by greenish colours with triangular symbols.

We generated developmental survival curves (which indicate changes of survival rate, i.e., percent survival, over developmental stages) for the six feeding groups (Figure 4). These survival curves showed stage-dependent changes of the percentage of the surviving individuals. The survival curves for the five groups examined in a previous study [7] were also included in the figure for comparison. Most of the curves were largely parallel with one another, and as a whole, these survival curves were statistically different from one another (*df* = 11, χ^2^ = 300, *p* < 0.0001, log rank test; *df* = 11, χ^2^ = 292, *p* < 0.0001, Wilcoxon rank test).

We noted that the Motomiya and Koriyama curves showed a great deal of mortality in the larva-prepupa period. The curves were segregated into three clusters of groups: a high survival cluster (Okinawa, Ube, Atami, Musashino, and Kashiwa), an intermediate survival cluster (Hirono and Motomiya), and a low survival cluster (Iitate-montane, Koriyama, Fukushima, and Iitate-flatland). These three clusters were statistically different from one another (*df* = 3, χ^2^ = 286, *p* < 0.0001, log rank test; *df* = 3, χ^2^ = 276, *p* < 0.0001, Wilcoxon rank test). However, the concentrations of caesium activity recorded in the leaves showed great variation within a given cluster. This intra-cluster variation most likely occurred because these groups were implemented using three different genetic lines of this butterfly species in different time periods, suggesting intraspecific variation regarding sensitivity to a radioactively contaminated diet. Accordingly, a set of groups that were assessed within the same time period were found to be comparable to one another as they come from the same genetic line (see Methods).

**Figure 5:**
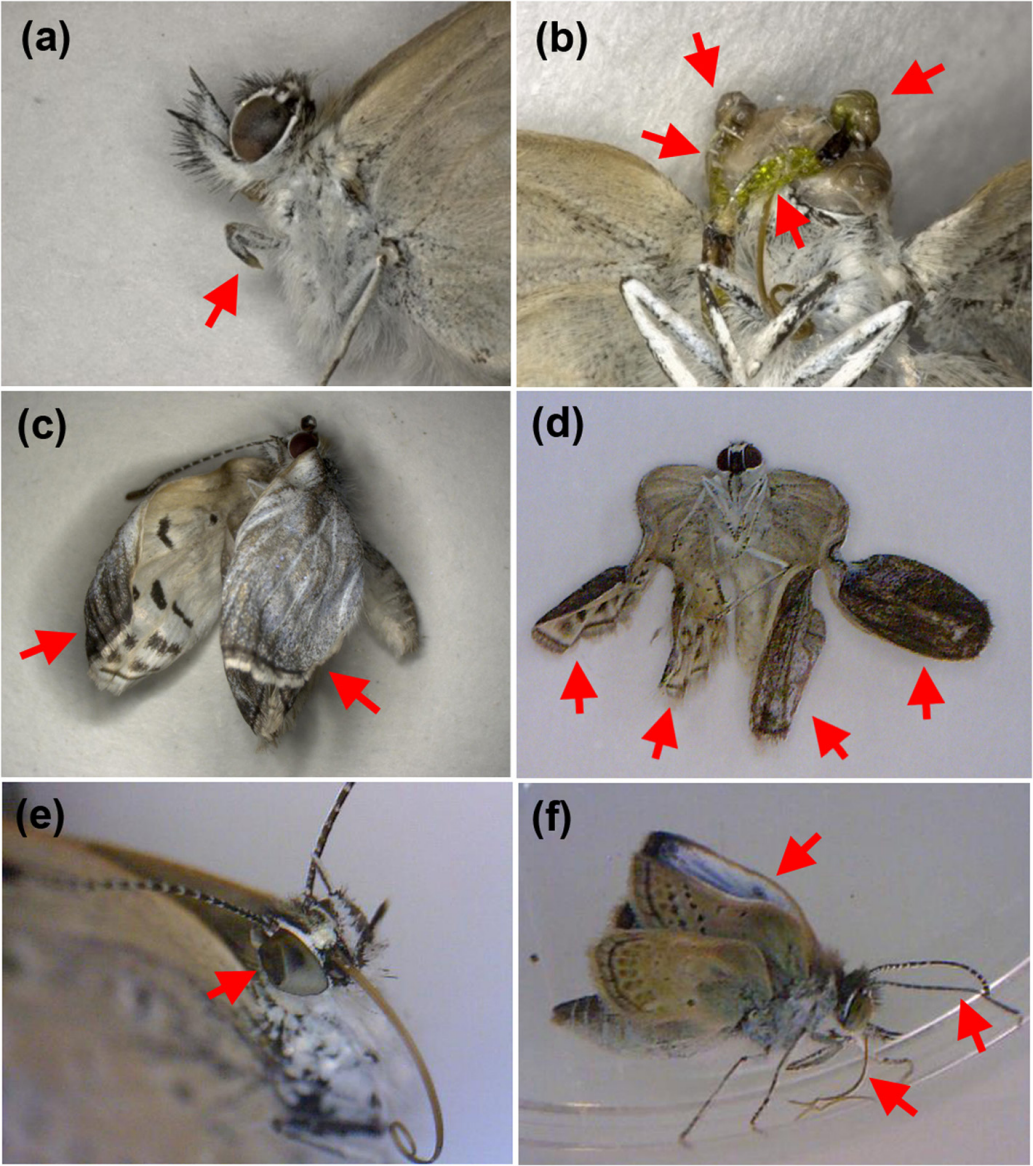
**Morphological abnormalities of F**_**1**_ **individuals.** Morphologically abnormal parts are indicated by red arrows. **(a)** Malformation of the left foreleg in an Atami sample. **(b)** Incomplete eclosion of the antennae in an Atami sample. **(c)** Curled wings in a Kashiwa sample. **(d)** Malformed wings in a Kashiwa sample. **(e)** Dented right compound eye in a Koriyama sample. **(f)** Abnormalities of the antennae, proboscis, and wings in a Motomiya sample.

We discovered various morphological abnormalities in the surviving adults (Figure 5). The severe and rare abnormalities shown in Figure 5 might imply the effects of a contaminated diet. Only three individuals in the group that consumed the control Okinawa leaves showed very minor morphological abnormalities, i.e., folded wings and a deformed leg, which were not comparably severe with the abnormal individuals from the other F_1_ groups.

### Effects on the F_2_ generation

**Figure 6:**
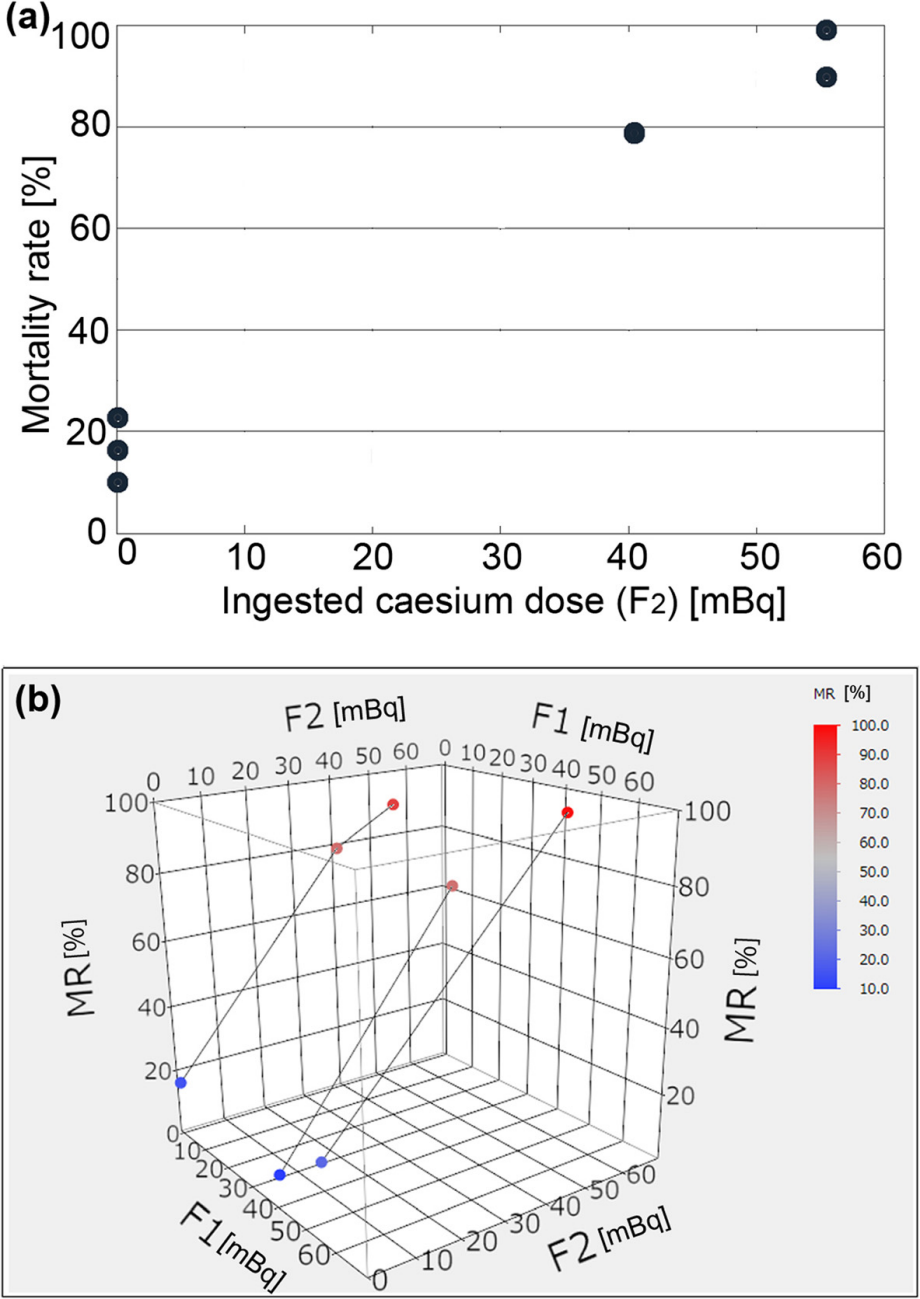
**Effects of the ingested caesium dose on the mortality rate in the F**_**2**_ **generation. (a)** A two-dimensional scatter plot. The ingested caesium dose in the F_2_ generation (but not the F_1_ generation) is provided on the *x*-axis. **(b)** A three-dimensional scatter plot. The ingested caesium doses in both the F_1_ and F_2_ generations are plotted. “F1” and “F2” indicate the ingested caesium dose in the F_1_ and F_2_ generations, respectively. “MR” indicates the mortality rate. Plots showing the same amount of F_1_ ingestion are connected by lines.

The F_2_ generation was obtained from the morphologically healthy (non-abnormal) F_1_ adults that consumed leaves from Okinawa, Koriyama, or Motomiya. Based on the detected caesium activity in the leaves, the ingested caesium doses were calculated (Table 4), together with the resultant mortality and abnormality rates (Table 5), and the dose–response relationship between the ingested dose and the mortality rate was examined. The mortality rate (*y*) appeared to be dependent on the caesium dose (*x*) ingested by the F_2_ generation with the regression model, *y* = 1.445 (±0.088) *x* + 17.04 (±3.24) (*R*^2^ = 0.982, *df* = 6, *F* = 267, *p* < 0.0001), but not significantly by the F_1_ generation (*R*^2^ = 0.00675, *df* = 6, *F* = 0.034, *p* = 0.86) (Figure 6a, b).

**Table 4:** **Ingested caesium dose in the F**_**2**_ **generation**

**F**_**2**_ **leaves from:**	**Number of larvae (** ***n*** **)**	**Ingested caesium dose [mBq]**
Okinawa	522 (=170 + 128 + 224)	0.0629 ± 0.0011
Koriyama	290 (=158 + 132)	40.4 ± 0.02
Motomiya	516 (=158 + 358)	55.4 ± 0.03

**Table 5:** **Mortality and abnormality rates in the F**_**2**_ generation* ^**1**^

**F**_**2**_ **leaves**	**Okinawa (F**_**2**_ **)**	**Koriyama (F**_**2**_ **)**	**Motomiya (F**_**2**_ **)**
**F**_**1**_ **leaves**			
Okinawa (F_1_)	*MR* = 16.5%;	*MR* = 79.1%;	*MR* = 89.9%;
	*AR* = 18.8%	*AR* = 79.7%	*AR* = 90.5%
	(*n* = 170)	(*n* = 158)	(*n* = 158)
Koriyama (F_1_)	*MR* = 10.2%;	*MR* = 78.8%;	Not tested.
	*AR* = 15.6%	*AR* = 87.9%	
	(*n* = 128)	(*n* = 132)	
Motomiya (F_1_)	*MR* = 22.8%;	Not tested.	*MR* = 99.2%;
	*AR* = 29.9%		*AR* = 99.2%
	(*n* = 224)		(*n* = 358)

*^1^ *MR*, mortality rate; *AR*, total abnormality rate; *n*, number of larvae examined.

**Figure 7:**
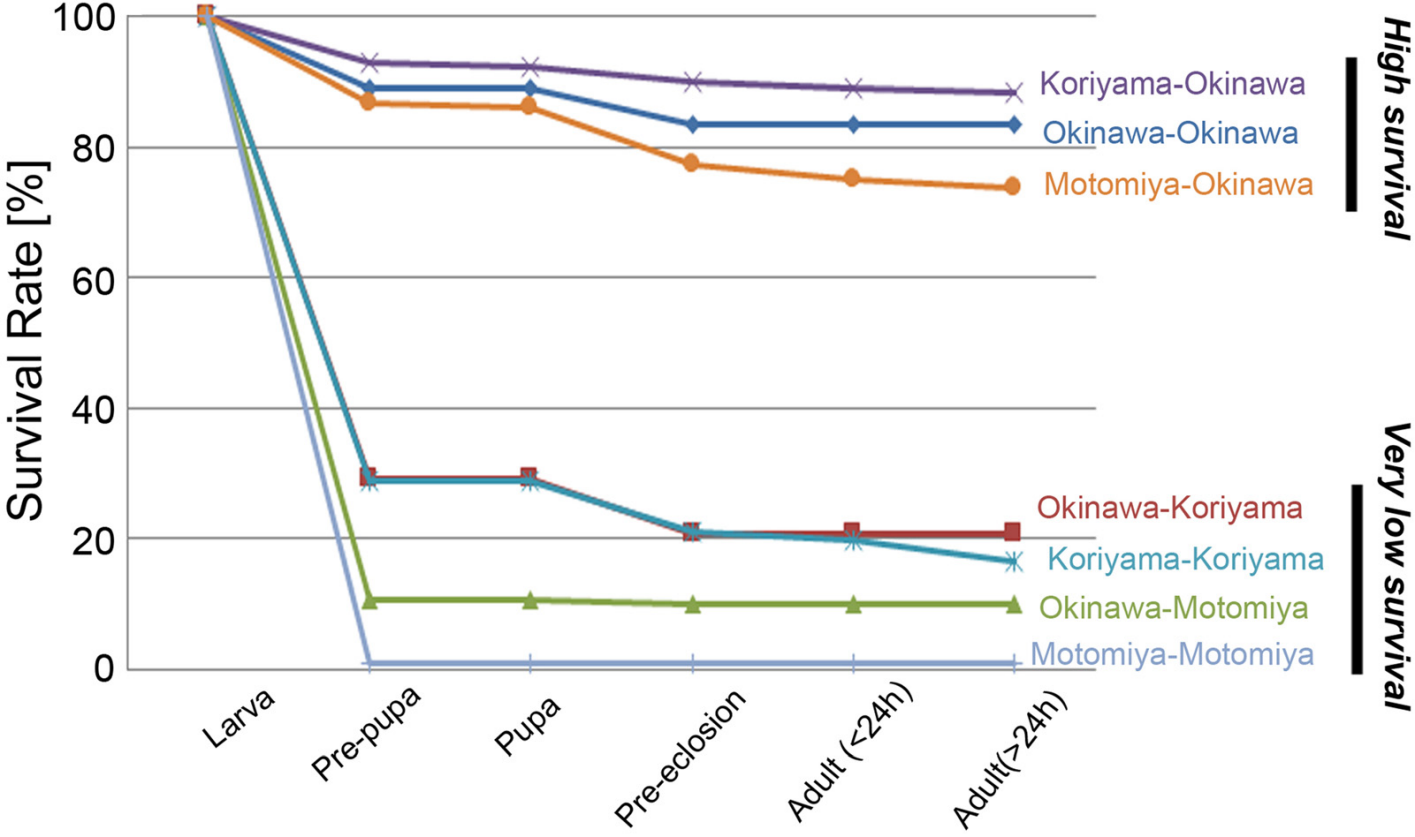
**Survival curves for the F**_**2**_ **generation.** The survival curves are segregated into two clusters of the experimental groups. For each curve, the localities of the F_1_ and F_2_ leaves are indicated, e.g., as Koriyama (F_1_)-Okinawa (F_2_).

Next, we produced developmental survival curves for the F_2_ generation (Figure 7). As a whole, these survival curves were highly different from one another (*df* = 6, χ^2^ = 818, *p* < 0.0001, log rank test; *df* = 6, χ^2^ = 824, *p* < 0.0001, Wilcoxon rank test). The curves were clearly segregated into two clusters: high and very low survival clusters. These two groups were statistically very different from each other (*df* = 1, χ^2^ = 758, *p* < 0.0001, log rank test; *df* = 1, χ^2^ = 774, *p* < 0.0001, Wilcoxon rank test). Again, it was apparent that the determinant of this segregation was the F_2_ leaves and not the F_1_ leaves. The remarkable segregation into the two clusters indicated the importance of the F_2_ diet.

**Figure 8:**
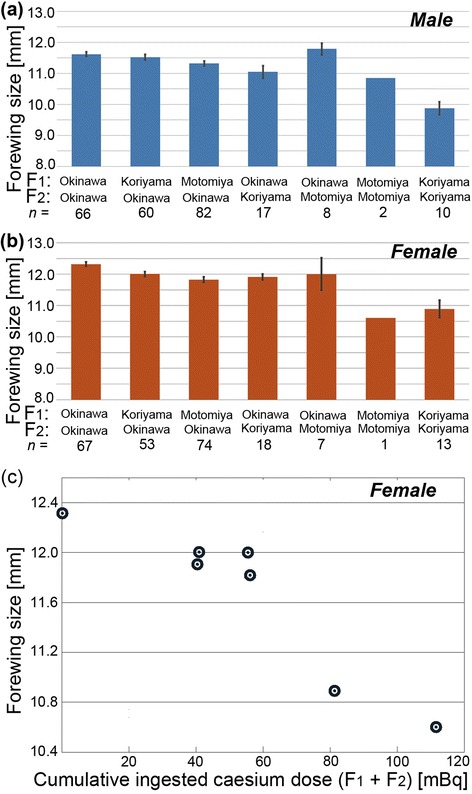
**Forewing size variation of the F**_**2**_ **generation. (a)** Male forewing size. Mean values are indicated, together with standard error bars. The Motomiya (F_1_)-Motomiya (F_2_) group showed a very small number of surviving adults, and thus, no error bars are shown for this group. These explanations are also applicable to the female graph shown in b. The difference between the Okinawa-Okinawa group and the Koriyama-Koriyama group is statistically significant (*p* < 0.0001, Student’s *t*-test). **(b)** Female forewing size. The difference between the Okinawa-Okinawa group and the Koriyama-Koriyama group is statistically significant (*p* = 0.0002, Welch’s *t*-test). **(c)** Relationship between the cumulative ingested caesium dose in the F_1_ and F_2_ generations and the forewing size. For the caesium dose, the total dose of ^137^Cs and ^134^Cs was used.

We compared the forewing sizes of the surviving adults (Figure 8a,b). The group that consumed the Koriyama leaves in both the F_1_ and F_2_ generations showed very small forewings in both sexes compared to the group that consumed the Okinawa leaves in both generations (*t* = 8.44, *df* = 74, *p* < 0.0001, Student’s *t*-test, for males; *t* = 5.03, *df* = 13, *p* = 0.0002, Welch’s *t*-test, for females). Interestingly, the cumulative ingested caesium dose throughout the F_1_ and F_2_ generations showed a higher correlation with the forewing size than the ingested dose in the F_2_ generation alone in both sexes, but especially in females (Table 6; Figure 8c). The female regression line between the cumulative ingested dose (*x*) throughout the F_1_ and F_2_ generations and the forewing size (*y*) was obtained as *y* = −1.70 (±0.31) *x* + 1.259 (±0.020) (*R*^2^ = 0.857, *df* = 6, *F* = 29.99, *p* = 0.0028).

**Table 6:** **Pearson correlation coefficient** ***r*** **and** ***p*** **-values for the F**_**2**_ **generation between the ingested caesium dose and forewing size**

**Caesium dose**	***r***	***p***
Male, F_2_ only	−0.35	0.44
Male, F_1_ + F_2_	−0.59	0.17
Female, F_2_ only	−0.58	0.17
Female, F_1_ + F_2_	−0.93	0.0028

**Figure 9:**
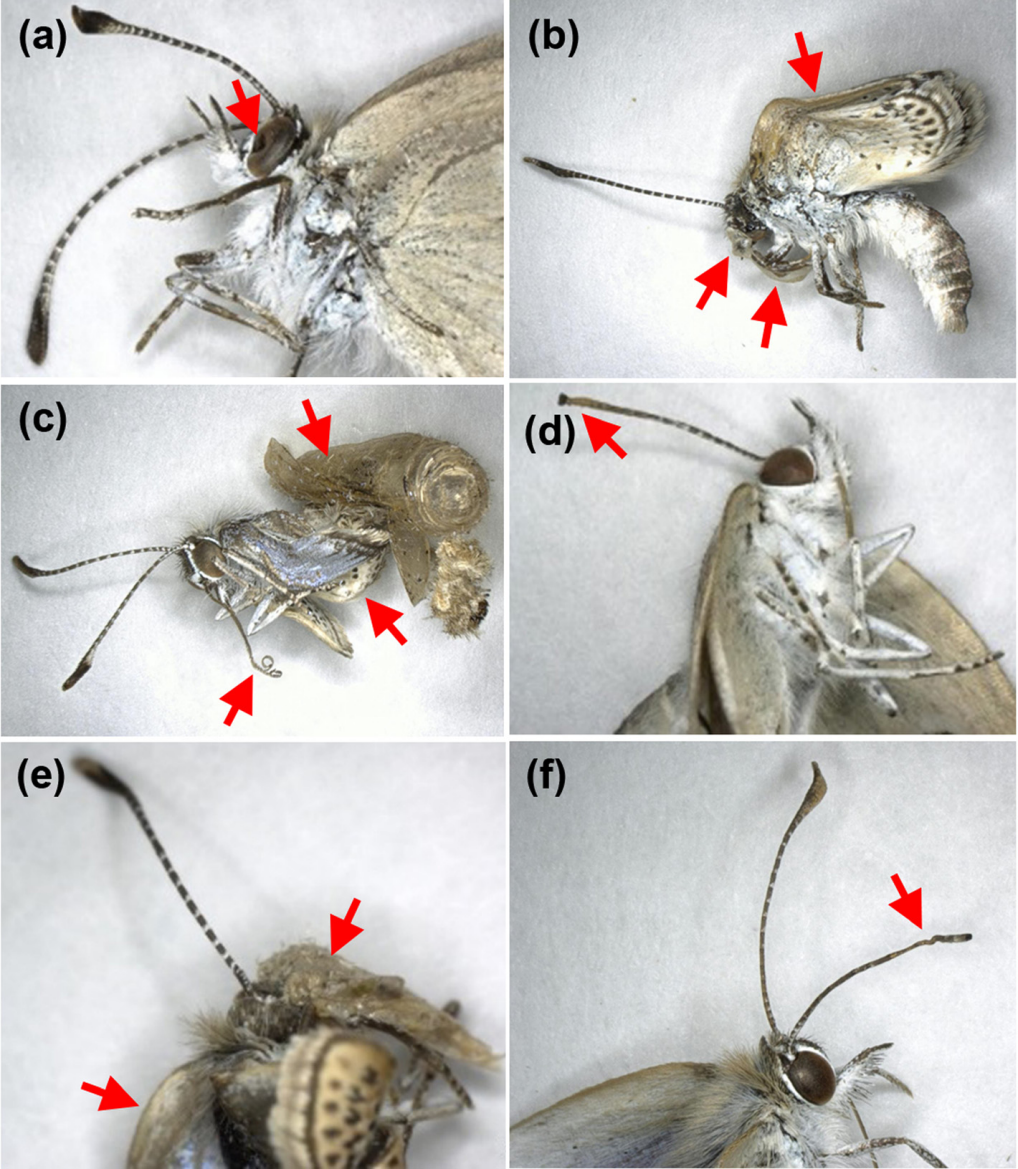
**Morphological abnormalities in F**_**2**_ **individuals.** Morphologically abnormal parts are indicated by red arrows. **(a)** Dented left compound eye in a Koriyama-Koriyama sample. **(b)** Eclosion failure in a Koriyama-Koriyama sample. **(c)** Eclosion failure in a Motomiya-Okinawa sample. **(d)** Truncated right antenna in a Koriyama-Okinawa sample. **(e)** Eclosion failure in an Okinawa-Motomiya sample. **(f)** Truncated right antenna in an Okinawa-Motomiya sample.

As observed in the F_1_ generation, various morphological abnormalities were detected in the surviving F_2_ adults (Figure 9). Considering the very high mortality and abnormality rates recorded, it is likely that many of the recorded effects are attributable to the contaminated diets ingested by the butterflies.

## Discussion

In the present study, we performed an internal exposure experiment using contaminated host plant leaves. The host plant leaves were collected from the localities showing low-level contamination, including two localities in Tohoku (Koriyama and Motomiya), two in Kanto (Kashiwa and Musashino), and one in Tokai (Atami). A similar experiment was performed previously [7], and the present study therefore serves to reproduce and validate the previous results. Furthermore, the present study extends the previous study by using the leaves that were less contaminated than those used in the previous study.

In the present study, low-dose effects were clearly detected in the butterflies in the form of high mortality and abnormality rates, low normality rates (robustness), low survival rates, and a small forewing size in the F_1_ generation of the Tohoku groups. It was somewhat surprising to find that the Koriyama group, which ingested 40.9 mBq of radioactive caesium during the larval stage from contaminated-contaminated leaves showing an activity of 117 Bq/kg, displayed mortality and abnormality rates greater than 50%, although the Motomiya group exhibited somewhat lower rates. These results suggest that low-dose ingestion of approximately 100 Bq/kg may be seriously toxic to certain organisms. Even among the surviving adults, a small forewing size was detected in the Koriyama and Motomiya groups, confirming the results concerning mortality rates, abnormality rates, normality rates, and survival rates.

For the Kanto and Tokai localities, which presented relatively low contamination levels ranging from 2.53 Bq/kg to 47.57 Bq/kg, both the mortality (survival) rates and the abnormality (normality) rates appeared to be dose dependent. These Kanto-Tokai groups were tested using the same genetic lines at the same time, which were comparable to one another. Therefore, the contaminated leaves from the Kanto-Tokai districts may have had small but detectable effects on the fitness of the butterflies. On the other hand, the forewing size of males in the Kanto-Tokai groups appeared to be larger than that of the Okinawa group, despite the possible adverse effects detected for other measures (Figure 3). The reasons for this size increase are not clear. The biological responses to very low doses may simply be different from the responses to relatively high doses.

Interestingly, the survival curves obtained for the different localities were segregated into three clusters: high, intermediate, and low survival clusters, not only among the groups from the previous study [7], but also in the groups of the present study, despite the large difference in caesium activity levels between the previous and present studies. In the previous study [7], the half mortality rate was calculated as 1.9 Bq per larva, corresponding to constant leaf consumption of 4.9 × 10^3^ Bq/kg throughout the larval stage. These previous values were more than 40 times as high as the values we obtained for Koriyama in the present study. As a result, within a given cluster of survival curves, the levels of leaf contamination were highly variable (see Figure 4). However, within a given set of groups that were treated within the same time period (hence, coming from the same genetic line), the survival rates were largely dependent on the caesium activity in the leaves. These results may originate simply from a dependence of radiation sensitivity on the genetic background of butterfly lines. That is, direct comparison between different butterfly lines may be difficult, even if the experimental set-ups are virtually identical with the exception of their genetic makeup. Therefore, our results suggest the existence of intraspecific individual variation of sensitivity to low-level radiation, which is consistent with the existing literature [13].

The biological effects of ingesting the contaminated diets were more severe in the F_2_ generation, despite the normal morphological appearance of their F_1_ parents. The differences can be illustrated as follows: The mortality rates of the Koriyama F_1_ group and the Koriyama F_2_ group obtained from the Koriyama F_1_ adults were 53.0% and 79%, respectively, while the mortality rates of the Motomiya F_1_ group and the Motomiya F_2_ group obtained from the Motomiya F_1_ adults were 31.2% and 99%, respectively. As the mortality rates of the Okinawa F_1_ group and the Okinawa F_2_ group obtained from the Okinawa F_1_ adults were 8% and 17%, respectively, the increase in mortality rates observed in the F_2_ generation cannot be readily attributed to the transgenerational accumulation of radiation effects. However, effects of the F_1_ generation on the F_2_ generation were discernable in the form of a small forewing size and its high correlation with the cumulative caesium dose, especially in females. It is important to note that a small forewing size can be induced not only through feeding of contaminated leaves, but also through long-term low-dose external irradiation in this species [7]. Furthermore, a small forewing size has been detected in field-caught butterflies from the polluted areas [7]. Therefore, the small forewing size observed in the present study implies that internal radiation exposure may have contributed to the high mortality rates recorded. Nevertheless, the F_1_ effect was small, and it could largely be overcome by the consumption of Okinawa leaves or amplified by the consumption of Koriyama or Motomiya leaves in the F_2_ generation. That is, the effects were transgenerational, but largely non-genetic physiological effects that could be overcome by dietary changes. Mechanistically, these outcomes may be explained by maternal effects, epigenetic effects, genomic instability, or similar phenomena.

These results potentially conflict with our previous findings showing that morphological abnormalities, including wing colour pattern aberrations, found in the F_1_ generation were heritable by the F_2_ generation [7-9]. In this previous experiment, we used P-generation adults collected from the field in the spring of 2011. These P-generation individuals were directly exposed to all of the radioactive materials from the explosion of the NPP when they were larvae, which could have resulted in genetic damage. We then employed morphologically abnormal F_1_ adults to obtain the F_2_ generation using non-contaminated leaves. In the case of the possible genetic damage indicated in 2011, the larvae may have consumed radioactive materials adsorbed on the surface of leaves. The contributions of β-ray-emitting nuclides in the earlier experiment cannot be ignored [14]. In contrast, the present study was performed in 2012, when the major radioactive species present was caesium, and the surface adsorption of radioactive materials was likely much less extreme. Therefore, the probability of genetic damage resulting from the ingestion of a contaminated diet was rather low in the present study. Both experimental set-ups are reasonable for examining heritable effects that are likely based on either genetic or physiological (or epigenetic) changes.

In the examination of forewing size, we noted a sex difference. Females showed a higher correlation with the ingested dose of radioactivity in both the F_1_ and F_2_ generations than males. The reasons for this sex difference are not clear. However, higher sensitivity of females to cold shock, a type of environmental stress, has been reported in this species of butterfly [4]. Hence, it is likely that females are generally more sensitive to stress than males of this species.

The internal exposure of organisms living in a polluted area through a contaminated diet is unavoidable. Indeed, the possible biological effects of contaminated diets on humans after the Chernobyl accident have been documented [15,16]. However, to the best of our knowledge, controlled experimental evaluations of internal exposure via a consumed diet are scarce in any organism in the field of radiation biology, with a few possible exceptions [17], although there may be some anecdotal or non-published studies that were performed following the Chernobyl accident. Thus, our experimental results constitute an important contribution to the research field. Accumulation of such scientific evidence is required to establish the low-dose biological effects of radiation [18].

## Conclusions

Our experimental system for detecting the biological effects of internal exposure to radiation via contaminated diets revealed that low-dose ingestion likely causes death (i.e., high mortality and low survival rates), disease (i.e., high abnormality and low normality rates), and growth retardation (i.e., a small forewing size), at least in some individuals of the pale grass blue butterfly. The effects of ingesting a contaminated diet on survival rates and forewing size were shown to be transgenerational. However, the effects of ingesting a contaminated diet in the F_1_ generation could be largely overcome by ingesting a non-contaminated diet in the F_2_ generation. It is likely that at least some of the biological effects of internal exposure to low doses of radiation are attributed to non-genetic physiological (or epigenetic) changes.

## Methods

### Ethics

No specific permissions were required to collect the pale grass blue butterfly *Z. maha* and its host plant of *O. corniculata* in Japan. The pale grass blue butterfly is the most common butterfly in Japan, and its host plant is a weed that is often a target for eradication in gardening and agriculture.

### Butterfly rearing

We followed standard rearing methods we have described previously [8], with a few minor modifications [4,7]. The egg collection procedure was also described elsewhere [7]. A pool of females was caught in the field in Okinawa (defined as the P generation) and used to collect eggs (defined as the F_1_ generation or the first offspring generation). These eggs were divided into four (in the summer of 2012) or three (in the fall of 2012) feeding groups in the F_1_ generation. Therefore, the genetic differences among these feeding groups could be ignored. Eggs were similarly obtained from a different pool of females caught in the field in Okinawa and were then divided into three feeding groups. These groups were fed leaves from Okinawa, Koriyama, or Motomiya. These additional three groups were employed in crosses with the first-generation groups to obtain the next generation (defined as the F_2_ generation or the second offspring generation). This process was necessary to avoid sibling crosses that could adversely affect the robustness of the offspring. To obtain the F_2_ eggs, morphologically normal and healthy (non-abnormal) adults were selected from one F_1_ group and mated with healthy adults from a different F_1_ group. In this paper, a group that consumed leaves from locality A in the F_1_ generation and leaves from locality B in the F_2_ generation is often indicated as the A-B group, but these butterflies all came from Okinawa lines genetically. All rearing experiments were performed in our laboratory, located on the Nishihara Campus of the University of the Ryukyus, Okinawa, except for the rearing of the Kanto-Tokai groups, which was carried out in our branch at Kichijouji, Musashino City, Tokyo, where the radiation level was maintained at 0.04-0.05 μSv/h, with the windows being closed at all times.

### Collection of host plant samples

Bunches of the host plant leaves of *O. corniculata* were collected from various localities in the summer and fall of 2012, as shown in Table 1. The bunches were covered with wet paper, stored in a chilled container, and sent to the laboratory, where they were stored in a refrigerator until use. The leaves stored in this way were still fresh when they were given to larvae. The bunches were stored separately, depending on the collection locality, to prevent cross contamination. Feeding experiments using the leaves from the Kanto and Tokai localities (Kashiwa, Musashino, and Atami) and Okinawa were performed in the summer of 2012, and feeding experiments using the leaves from the Tohoku localities (Koriyama and Motomiya) and Okinawa were performed in the fall of 2012.

### Forewing size measurement

We used an SKM-2000 digital microscope and the associated software SK Measure (Saito Kougaku, Yokohama, Japan) to measure forewing size in digital images. We measured the distance from the base to the marginal band at the end of the M_1_ vein on the ventral side. Because the feeding experiments involving leaves from the Kanto and Tohoku localities were performed separately, the Okinawa groups for the summer and fall experiments were both set at 1.00 in Figure 3, so that their results could be shown in a single graph.

### Measurement and calculation of radioactivity

The ground radiation dose at the collection sites was measured using an Aloka TCS-161 scintillation survey meter (Hitachi Aloka Medical, Tokyo, Japan) or an RAE Systems DoseRAE 2 dosimeter (San Jose, CA, USA) on the surface of the ground (0 cm high). Radioactivity measurements of caesium in the host plant and pupal samples were performed using a Canberra GCW-4023 germanium radiation detector (Meriden, CT, USA) at the Instrumental Research Center of the University of the Ryukyus. Measurements were also performed using an Ortec GMX30 N-type HP germanium detector (Oak Ridge, TN, USA) at Nagasaki University to check the reproducibility of the measurements. The host plant samples were dried and burned to ashes (flameless combustion) before being subjected to measurements, as described previously [7,11]. Pupal samples were measured in a small columnar plastic container as indicated in a previous report [11], but the results were all below the detection limit.

To calculate the amount of caesium ingested, we assumed that the larvae hatched on the sixth day following egg deposition, and they were subsequently fed Okinawa leaves for eight days, after which contaminated leaves from the different localities were provided. Thus, for the first 14 days after egg deposition, all larvae consumed Okinawa leaves. Based on previous data [7], we estimated that approximately 10% of the entire amount of leaves that a larva consumed came from Okinawa leaves, which was taken into account to calculate the ingested caesium dose. Furthermore, for simplicity, we assumed that the larvae ate all of the leaves required for subsequent growth at once on the first day of ingestion of the contaminated leaves and that ^137^Cs and ^134^Cs were released at a 1:1 activity ratio on 15 March 2011 in a single burst from the Fukushima Dai-ichi NPP.

### Statistical analysis and graphics

We used the statistical software R version 3.0.2 (R Foundation for Statistical Computing, Vienna, Austria), JSTAT version 13.0 (Yokohama, Japan), and JMP 11.0.0 (2013) (SAS Institute, Cary, NC, USA). Linear regression equations were expressed with standard error and accessed with *R*^2^ and *p*-values. Data were also evaluated with Pearson correlation coefficient *r*. To assess forewing size differences, either Student’s or Welch’s *t*-test was performed, in accordance with *F*-test. Survival curves were examined by log rank test and Wilcoxon rank test. Data were graphically presented using Excel (2013) (Microsoft).

## References

[1] Møller AP, Hagiwara A, Matsui S, Kasahara S, Kawatsu K, Nishiumi I, Suzuki H, Ueda K, Mousseau TA (2012). Abundance of birds in Fukushima as judges from Chernobyl. Environ Pollut.

[2] Møller AP, Nishiumi I, Suzuki H, Ueda K, Mousseau TA (2013). Differences in effects of radiation on abundance of animals in Fukushima and Chernobyl. Ecol Indicat.

[3] Akimoto S (2014). Morphological abnormalities in gall-forming aphids in a radiation-contaminated area near Fukushima Daiichi: selective impact of fallout?. Ecol Evol.

[4] Otaki JM, Hiyama A, Iwata M, Kudo T: **Phenotypic plasticity in the range-margin population of the lycaenid butterfly*****Zizeeria maha*****.** *BMC Evol Biol* 2010, **10:**252. 10.1186/1471-2148-10-252 PMC2931505 20718993

[5] Buckley J, Bridle JR, Pomiankowski A (2010). Novel variation associated with species range expansion. BMC Evol Biol.

[6] Hiyama A, Taira W, Otaki JM (2012). Color-pattern evolution in response to environmental stress in butterflies. Front Genet.

[7] Hiyama A, Nohara C, Kinjo S, Taira W, Gima S, Tanahara A, Otaki JM (2012). The biological impacts of the Fukushima nuclear accident on the pale grass blue butterfly. Sci Rep.

[8] Hiyama A, Nohara C, Taira W, Kinjo S, Iwata M, Otaki JM (2013). The Fukushima nuclear accident and the pale grass blue butterfly: evaluating biological effects of long-term low-dose exposures. BMC Evol Biol.

[9] Taira W, Nohara C, Hiyama A, Otaki JM (2014). Fukushima’s biological impacts: the case of the pale grass blue butterfly. J Hered.

[10] Bauerfeind SS, Fischer K (2005). Effects of food stress and density in different life stages on reproduction in a butterfly. Oikos.

[11] Nohara C, Hiyama A, Taira W, Otaki JM (2014). The biological impacts of ingested radioactive materials on the pale grass blue butterfly. Sci Rep.

[12] Hiyama A, Iwata M, Otaki JM: **Rearing the pale grass blue*****Zizeeria maha*****(Lepidoptera, Lycaenidae): toward the establishment of a lycaenid model system for butterfly physiology and genetics.** *Entomol Sci* 2010, **13:**293–302.

[13] Møller AP, Mousseau TA (2013). The effects of natural variation in background radioactivity on humans, animals and other organisms. Biol Rev.

[14] Endo S, Tanaka K, Kajimoto T, Thanh NT, Otaki JM, Imanaka T (2014). Estimation of β-ray dose in air and soil from Fukushima Daiichi Power Plant accident. J Rad Res.

[15] Lüning G, Scheer J, Schmidt M, Ziggel H (1989). Early infant mortality in West Germany before and after Chernobyl. Lancet.

[16] Gould JM, Sternglass EJ (1989). Low-level radiation and mortality. Chemtech.

[17] Cahill DF, Wright JF, Godbold JH, Ward JM, Laskey JW, Tompkins EA: **Neoplastic and life-span effects of chronic exposure to tritium. II. Rats exposed*****in utero*****.** *J Natl Cancer Inst* 1975, **55:**1165–1169. 10.1093/jnci/55.5.1165 1206742

[18] Møller AP, Mousseau TA (2013). Low-dose radiation, scientific scrutiny, and requirements for demonstrating effects. BMC Biol.

